# Interfacial Phenomenon and Nanostructural Enhancements in Palladium Loaded Lanthanum Hydroxide Nanorods for Heterogeneous Catalytic Applications

**DOI:** 10.1038/s41598-018-22800-0

**Published:** 2018-03-12

**Authors:** Ammar Bin Yousaf, Muhammad Imran, Muhammad Farooq, Peter Kasak

**Affiliations:** 1 Center for Advanced Materials, Qatar University, Doha, 2713 Qatar; 2 Hefei National Laboratory for Physical Sciences at Microscale, University of Science and Technology of China, Hefei, Anhui 230026 P.R. China; 3 Department of Chemistry, University of Management and Technology, Lahore, 54000 Pakistan

## Abstract

Hydrogenation and cross-coupling reactions are of great importance for industrial applications and noble metal based catalysts are filling the void since the last few decades. However, the high cost of noble metals and poor recycling performance provides an opportunity for chemists to look for alternate options. Herein, we present the use of Lanthanum hydroxide as support for loading ultra-low amount of Pd for hydrogenation and cross-coupling reactions. Lanthanum hydroxide having controlled morphologies comprises exposed crystallographic facets which interact with small sized Pd NPs and shows versatile and effective catalytic performance. The reduction of 4-NP over Pd/La(OH)3 was achieved within very short time (45s) with a rate constant of 60 × 10^−3^ s^−1^. The hydrogenation of styrene was also accomplished within 1 hour with much high TOF value (3260 h^−1^). Moreover, the Suzuki cross-couplings of iodobenzene and phenyl boronic acid into biphenyl completed within 35 min with a TOF value of 389 h^−1^. The strong interfacial electronic communication regulates electron density of catalytic sites and lowers energy for adsorption of reactant and subsequently conversion into products. Moreover, abundant hydroxyl groups on the surface of La(OH)3, large surface area, mono-dispersity and ultra-small size of Pd NPs also favors the efficient conversion of reactants.

## Introduction

Heterogeneous catalysis of hydrogenation and cross-coupling reactions are important avenue that find wide industrial application in the production of fine chemicals, agrochemicals, fragrances, flavors, pharmaceuticals and dietary supplements^1,2^. A large number of noble metal based catalysts have been introduced in the last decade as cross-coupling and hydrogenation catalysts. However, high cost of noble metals, aggregation of nanoparticles due to high surface energy, poor recycling performance and high yields of products usually achieved at relatively high temperatures forced researchers to find alternate options^3,4^. Ultra low amount of noble metals supported on various supports have shown promising potential^5–8^. Moreover, the interfacial interactions of Pd with the support often control the performance of the catalyst for organic conversions and can be tuned for new and enhanced functions. The selectivity and sensitivity of reactions can also be enhanced through the use of electrostatics interactions, regulating electron density of catalytic sites and controlling the size of metal nanoparticles. Moreover, the interfacial interaction of Pd loaded MnO_*x*_–CeO_2_–C hetero-nanostructure also showed better performance for hydrogenation reactions^9^.

Recent years have witnessed considerable interest of rare earth oxides and hydroxides in catalysis^10,11^ luminescent devices, communication^12^ time resolved fluorescence (TRF) labels for biological detection^13,14^ and many others applications on account of their unique optical, electronic, magnetic and chemical properties arising from their 4 *f* electrons^15–17^. Lanthanum oxide have been used for oxidation of methane^18,19^, methane dry reforming^20^ and trans-esterification reactions^21,22^. However, Lanthanum oxide (La_2_O_3_) is highly hygroscopic in nature and readily converts to Lanthanum hydroxide under atmosphere conditions. Lanthanum hydroxide La(OH)_3_ is also an important basic hydroxide and have been used for heterogeneous catalysis for a number of applications, however its potential as support in organic transformations is still unexplored^23–25^. Lanthanum hydroxide has controlled morphologies that comprise preferentially exposed crystallographic facets which show more versatile and effective performance^26^.

Herein, we present a unique strategy for the synthesis of strongly coupled Pd NPs with lanthanum hydroxide rods for the reduction of 4-nitrophenol into 4-aminophenol, hydrogenation of styrene and Suzuki cross-coupling reactions^27^. The abundant hydroxyl groups on lanthanum hydroxide offers plenty of anchoring sites for Pd ion and subsequently immobilizing Pd NPs resulting in the confinement of NPs and minimizing aggregation. The as synthesized hexagonal La(OH)_3_ rods have highly isotropic structure along the *c*-axis and interacts with the planes of Pd resulting in exposed catalytic centers for hydrogenation and cross coupling reactions.

## Results and Discussion

**Figure 1:**
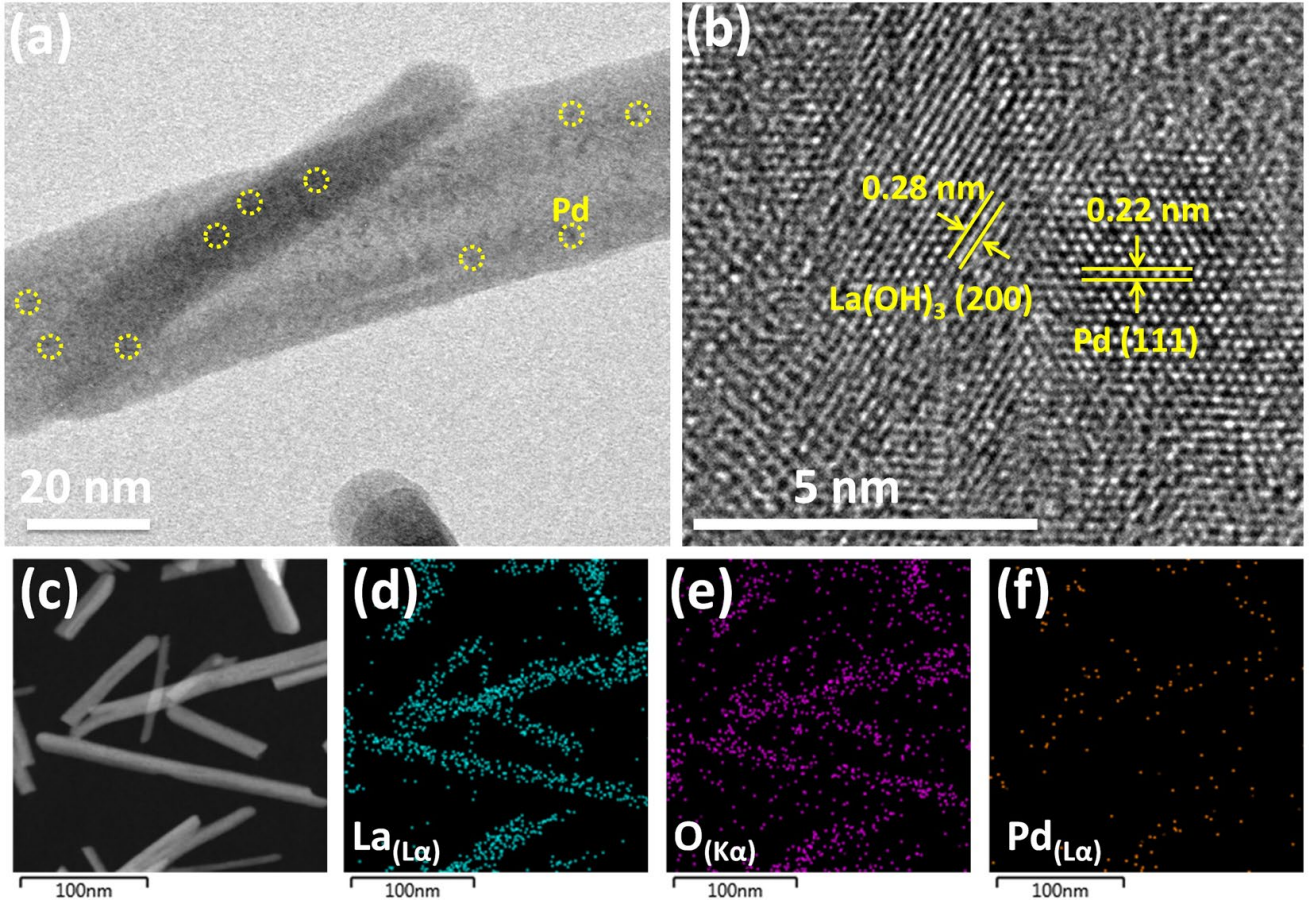
TEM image (**a**), HRTEM image (**b**) and HAADF-STEM element mappings of Pd/La(OH)_3_ nanocatalyst (**c–f**).

The morphology of the as-synthesized products was observed through scanning electron microscopy (SEM) and transmission electron microscopy (TEM). Typical SEM and TEM image of Lanthanum hydroxide nanorods are displayed in Figures S1 and S2. It can be seen clearly that La(OH)_3_ mainly consists of numerous well-defined nanorods with length ranging in several hundreds of nanometers and width of about 20–50 nm. The low magnification TEM image of Pd/La(OH)_3_ shows the dark colored tiny dots of Pd NPs which are about 2–3 nm in size and clearly suggests the nanostructure was successfully prepared. (Fig. 1a)

**Figure 2:**
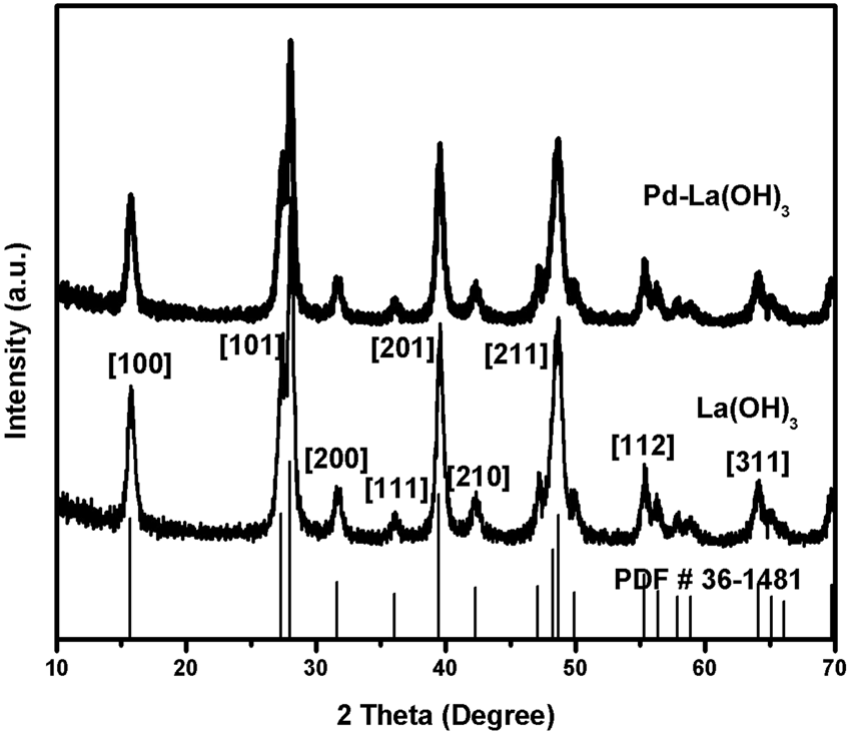
XRD patterns of La(OH)_3_ nanorods and Pd/La(OH)_3_ nanocatalyst.

The high magnification TEM image (Fig. 1b) reveal that La(OH)_3_ nanorods are clearly faceted and the apparent lattice fringes indicate the high crystallinity. The calculated distance (0.28 nm) between the adjacent lattice fringes corresponds well to the [200] plane of pure hexagonal Lanthanum hydroxide (JCPDS No. 36–1481). The HRTEM image also reveals Pd nanoparticles loaded onto the surface of La(OH)_3_ with observed lattice spacing value of 0.22 nm ascribed to the [111] plane of Pd. The structure of lanthanum hydroxide nanorods belongs to hexagonal *P6*_3_*/m* in which the unit cell exhibits two molecules of La(OH)_3_ and a La atom situated inside the trigonal prism having infinite linear chain which are parallel to the *c*-axis^26^. The hexagonal La(OH)_3_ has a highly isotropic structure along the c-axis and interacts with the plane of Pd having facets interaction.(Fig. 2b)

This interfacial phenomenon results in strong metal-support interaction which is also evident from XPS analysis. In addition, HAADF-STEM element mappings were also taken to strengthen the claim of successful loading of Pd NPs onto La(OH)_3_ nanorods. It can be clearly seen that Pd is evenly and homogeneous distributed onto the nanorods indicating the high dispersity of nanoparticles. (Fig. 1c–f) We have also performed the Zeta potential measurements of La(OH)_3_ nanorods to check the surface electric potential. The zeta potential value appeared to be −23.5 mV, clearly suggesting that the abundant hydroxyl groups on surface of La(OH)_3_ have negative charge which facilitate the interaction of positive Pd ions, thus preventing the aggregation of Pd nanoparticles.

XRD patterns of as synthesized La(OH)_3_ and Pd/La(OH)_3_ are shown in Fig. 2. It is obvious that both samples are high crystalline and the diffraction peaks corresponding to the crystal planes of (100) (15.6°), (101) (27.9°) and (111) (36.0°) are readily assigned to lanthanum hydroxide having JCPDS No. 36-1481^28^. The lattice parameters are a = 6.529Å b = 6.529 Å and c = 3.859Å with hexagonal symmetry and space group of *P6*_3_*/m*. The peaks from other phases were not detected which indicates the high purity of the samples. In case of Pd-La(OH)_3_, no obvious Pd signals were observed due to ultra low (actual weight content: 0.98 wt% determined by ICP-MS) loadings and very small size of Pd nanoparticles. (Table S3)

**Figure 3:**
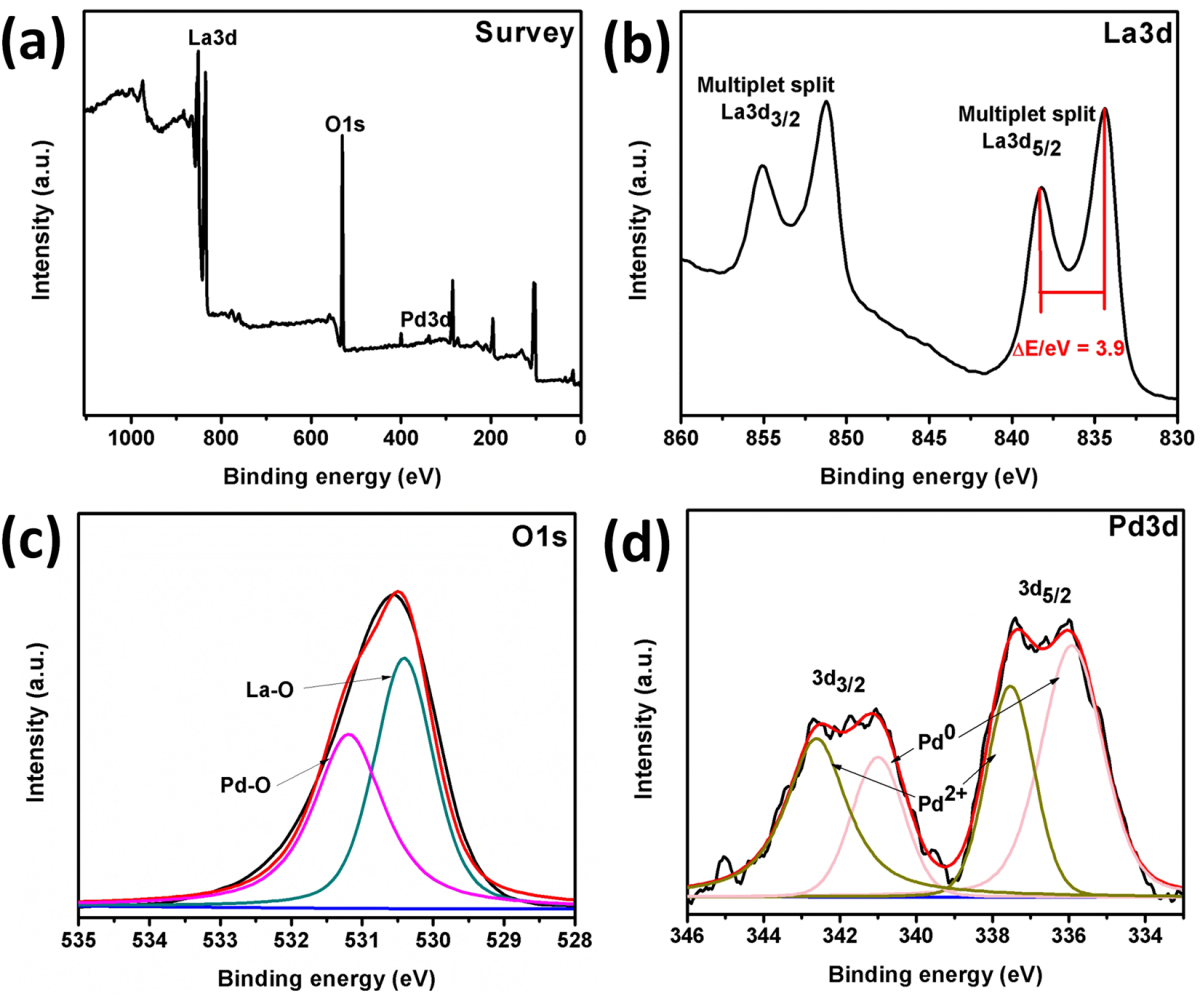
Survey XPS spectrum of Pd/La(OH)_3_ nanocatalyst (**a**), High resolution XPS spectra of La3d (**b**), O1s (**c**) and Pd3d (d) orbitals.

X-ray photoelectron spectroscopy (XPS) analysis was used to evaluate the surface composition and valence states of Pd-La(OH)_3_. The representative XPS survey scan spectrum (Fig. 3a) indicates the existence of La, O and Pd elements. Figure 3b exhibits the La3d binding energy (830–860 eV) region with La3d_5/2_ is at 834.3 eV and La3d_3/2_ exhibit binding energy of 851.0 eV. The La3d region has spin-orbit components and each spin-orbit component is further split by multiplet splitting. La(OH)_3_ shows four components and the intensity ratio of La3d_5/2_ is found to be DE/eV = 3.9, which is characteristic to Lanthanum hydroxide^29^. The corresponding O 1s spectrum is displayed in Fig. 3c, the O 1s spectrum is broad and asymmetric and can be deconvoluted into two peaks, indicating the presence of oxygen bonded to lanthanum (La-O) at binding energy 530.3 eV, and Pd (Pd-O) at binding energy of 531.2 eV^30^. The high-resolution XPS spectrum of the Pd 3d region (Fig. 3d) reveals the existence of metallic Pd(0) and Pd (II) with binding energies at 3d_5/2_ = 335.7 eV, 337.5 eV and 3d_3/2_ = 340.9 eV, 342.6 eV respectively.

**Figure 4:**
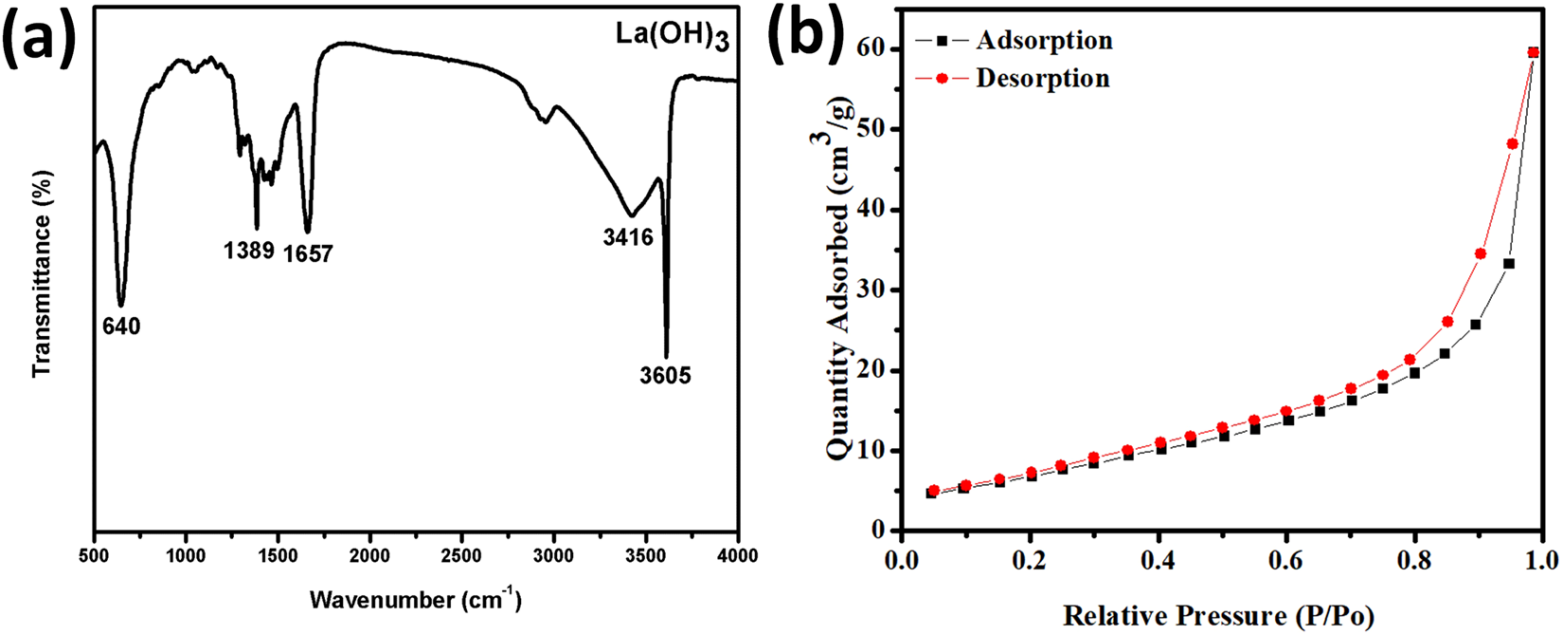
FTIR spectra (a) and N_2_ adsorption-desorption isotherm of La(OH)_3_ nanorods.

The abundant hydroxyl groups on lanthanum hydroxide offers plenty of anchoring sites for Pd ion and subsequently immobilizing Pd NPs resulting in the confinement of NPs and minimizing aggregation. It can be clearly seen that the existence of Pd (II) demonstrate that Pd have strong bonding with -OH groups present on the surface of La(OH)_3_^31^. FTIR spectrum of the La(OH)_3_ shown in Fig. 4a reveals a sharp band appearing at 3605 cm^−1^ which is attributed to the bulk hydroxyl groups originating from La(OH)_3_ while the peak at 3416 cm^−1^ is due to the surface adsorbed water molecules. The weak intensity peaks at 1657, 1389, 640 cm^−1^ are mainly due to the stretching vibrations from La-OH and water adsorbed molecules^32^. The specific surface area of lanthanum hydroxide nanorods was also measured by nitrogen adsorption-desorption isotherms.(Fig. 4b) Nitrogen adsorption-desorption experiment was carried out at 77 K using N_2_. The BET specific surface area of lanthanum hydroxide nanorods was observed to be 48 m^2^/g, which favor the dispersion of Pd nanoparticles, adsorption of reactants and subsequently present them at the interface of metal-support for conversion into products.

To examine the catalytic performance of as-synthesized Pd loaded lanthanum hydroxide, the reduction of 4-nitrophenol (NP) to 4-aminophenol (AP), hydrogenation of styrene and Suzuki cross-coupling reactions were chosen as model reactions. The reduction of 4-NP to 4-AP was carried out in the excess of sodium borohydride, where NaBH_4_ first dissociate in aqueous solution and produces borohydride ions which interacts with the Pd NPs and transfer hydrogen species to the host which then reacts with the 4-NP to convert it into 4-AP^33^. The reduction of 4-NP to 4-AP was done by adding 4-NP (60 μL, 10 mM) to 7 mL of NaBH_4_ aqueous solution (0.1 M) and 1 mL of catalyst dispersion (0.1 mg mL^−1^). (see Experimental section)

**Figure 5:**
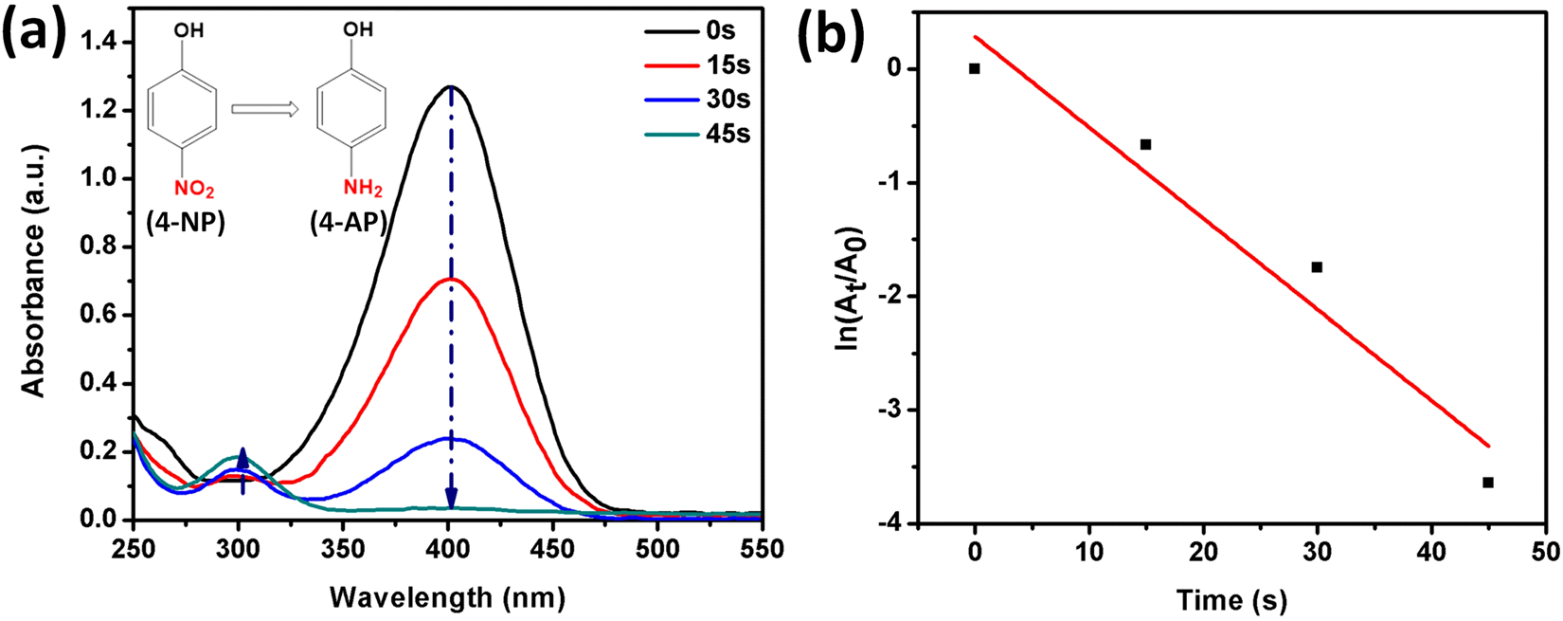
Time dependent UV-Visible spectra of 4-NP reduction over Pd/La(OH)_3_ nanocatalyst (**a**), Plot of ln(A_t_/A_0_) against the reaction time (**b**).

The original 4-nitrophenol aqueous solution shows a absorption peak at 317 nm which shifted to 400 nm after the addition of NaBH_4_ indicating the formation of 4-nitrophenolate ion and color of the solution changed from light yellow to bright yellow color. (Figure S3) After the addition of Pd/La(OH)_3_ catalyst dispersion into 4-NP and NaBH_4_ aqueous solution, the colour of the mixture changes from bright yellow to colourless and the peak at 400 nm in UV-visible spectra also gradually reduced and a new peak emerges at 298 nm. (Fig. 5a) The results showed complete reduction of 4-NP within 45 seconds by using Pd/La(OH)_3_ as catalyst which exhibit considerably high catalytic activity compared to other reports.(Table S1) The bare La(OH)_3_ nanorods does not show any activity even after 20 min of reaction time clearly suggesting the enhanced role of Pd NPs. (Figure S4) The mechanistic reason behind this improved activity is considered to the mono-dispersity and ultra small size of Pd NPs. The interfacial interaction of Pd and La(OH)_3_ nanorods as supported by HRTEM images, also favours the catalytic performance as the reduction ability greatly depends on the transference of electrons within the system. The electronic interaction between Pd NPs and abundant -OH groups present on the surface of lanthanum hydroxide as evident from XPS analysis synergistically enhanced the reduction of 4-NP. The borohydride ions react with Pd NPs and at the same time 4-NP also adsorbed on the surface of lanthanum hydroxide and present at the interface of metal-support and efficiently reduces nitrophenol to the nitroso compound, hydroxyl amine and finally to the 4-aminophenol^27,34^. The strong interfacial interaction and fast electronic communication results in an overall increase of catalytic performance. The kinetics of the 4-NP reduction into 4-AP was also calculated as a function of time by taking absorbance maximum at 400 nm. As the amount of NaBH_4_ was in access throughout the course of reaction, the pseudo-first order kinetic can be applied^35^. A linear relationship is obtained between *ln*(A_t_/A_0_) and reaction time as shown in Fig. 5b. The rate constant value obtained to be 60 × 10^–3^ which is much higher than previous reports. (Table S1) Moreover, the recyclability of the catalyst was also observed as it is an important aspect for practical applicability.

The recyclability tests were performed by removing the catalyst from reaction mixture after first run washed it with water and ethanol and drying in vacuum oven. The recycled catalyst was then used for each run under similar conditions. As shown in Figure S3, the as-synthesized Pd/La(OH)_3_ nanorods exhibit excellent performance and durability slightly decreased even after 5 consecutive cycles. (Figure S5) The enhanced and durable performance of our catalyst makes it a suitable candidate for industrial and other applications.

**Figure 6:**
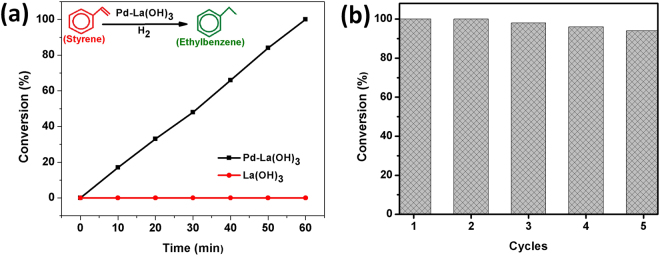
Hydrogenation of styrene to ethylbenzene over Pd/La(OH)_3_ (a) and Recycling performance of Pd/La(OH)_3_ for styrene hydrogenation.

The hydrogenation of styrene into ethylbenzene was also chosen as model reaction using H_2_ as reductant. The hydrogenation reactions are of great importance in industry to convert liquid vegetable oil into margarine and other valuable products and an efficient catalyst is of great need^36^. The hydrogenation of styrene by as-synthesized Pd/La(OH)_3_ catalyst was carried out in absolute ethanol using H_2_ as the reductant at room temperature.(see Experimental section) As shown in the Fig. 6a, the Pd loaded Lanthanum hydroxide showed a complete conversion of styrene into ethylbenzene in just 60 min while the bare La(OH)_3_ nanorods does not exhibit any activity clearly suggesting the active role of ultra small amount of Pd for catalytic conversion. The actual weight content of Pd was calculated to 0.98% by ICP-MS analysis and the turnover frequency for Pd/La(OH)_3_ (the moles of product formed per moles of noble metal per hour) was calculated to 3260 h^−1^ which is much higher than previously reported data. (Tables S2, S3) The enhanced catalytic performance clearly suggests that the adsorption and conversion of reactants into products is greatly favored by the catalyst. The molecular hydrogen readily attach on the Pd NPs and forms metal hydride while abundant hydroxyl groups, outer *f*-orbital electrons of Lanthanum and increased surface area as evident from BET favors the adsorption of reactants and present them at the interface for subsequent conversion^27,37,38^. The electronic communication between Pd NPs and Lanthanum hydroxide nanorods provides greater stability, reduces the energy required to dissociate H-H bond and double bond of styrene readily converts into single bond consequently improving the overall catalytic activity^39^. The recyclability of catalyst for industrially applicability was also examined by using the same catalyst under similar conditions. As shown in Fig. 6b, the catalyst showed minimal reduction in activity after 5 continuous cycles, clearly suggest that our as-synthesized catalyst exhibit excellent performance for the hydrogenation reactions. To evaluate the morphology and crystal phase of used catalyst, we performed the SEM and XRD measurements. The results reveal that the morphology and crystal phase of Pd/La(OH)_3_ nanorods were well maintained after recycling tests. The corresponding data has been provided in supporting information (Figures S6, S7).

The formation of *sp*^2^-carbon bonds by cross-coupling reactions is also an important reaction for the synthesis of fine chemicals and large scale industrial products^40^. Pd based homogenous catalyst have been reported so for the Suzuki cross-coupling reaction, however, the difficulty in separation of end products is a daunting challenge and provide an opportunity for chemist to look for other heterogeneous catalyst. We have also used the as-synthesized Pd/La(OH)_3_ catalyst for potential use in Suzuki cross-coupling reactions. The cross-coupling reaction was carried out at 65 °C in ethanol:water (8:2 volume ratio) reaction solvent. (see Experiment section)

**Figure 7:**
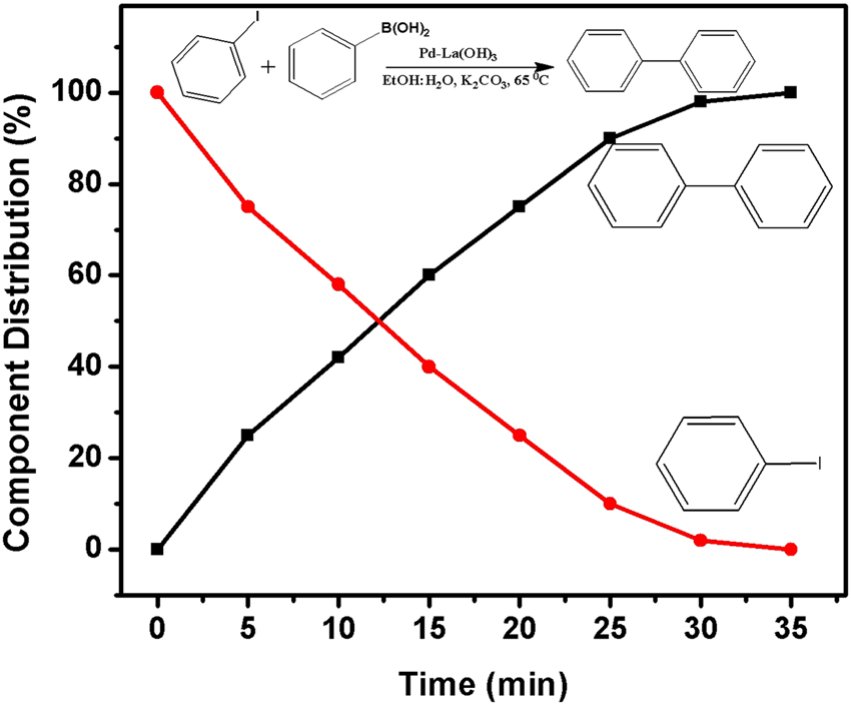
The conversion % of iodobenzene and the yields of biphenyl as a function of time.

As shown in Fig. 7, the coupling of phenylboronic acid and iodobenzene converts into biphenyl within just 35 min with 100% selectivity. The catalytic performance of Pd/La(OH)_3_ was evaluated by calculating the turnover frequency TOF (the moles of iodobenzene/ mole of Pd catalyst per hour), resulting in much higher value of 389 h^−1^.

The mechanistic investigation of the enhanced performance could be explained as, the Pd NPs act as reaction centers and support plays a role as supporter. The rate-determining step for Suzuki cross-coupling reaction generally is the dissociation of the C−I bond and the outer 4 *f* electron of Lanthanum readily act as electron-donor to increase electron density on the palladium *d*-band center^41^. The adsorption of reactants is also favored by the abundant hydroxyl groups (Brønsted sites) which act as hydrophilic points for the adsorption of reactants. Moreover, these hydroxyl groups also provide additional intermolecular forces such as hydrogen-bonding between the support and reactants thus favoring the adsorption rate and efficiency. Moreover the strong electronic communication between and support and Pd also enhances the catalytic performance. Our results offer a new strategy for nanostructural design of interracially interacted highly efficient heterogeneous catalysts for organic transformations.

## Conclusions

In conclusion, we have successfully synthesized highly efficient nanocatalyst for the hydrogenation and cross-coupling reactions. The as-synthesized Pd/La(OH)_3_ nanocatalyst was characterized by SEM, TEM, HRTEM, XRD and elemental mappings. The catalyst showed enhanced performance for the reduction of 4-NP to 4-AP with a rate constant of 60 × 10^−3^ s^−1^. The styrene hydrogenation was also achieved with 60 min with a TOF value of 3260 h^−1^. Moreover, the Suzuki-corss coupling reaction was also studied by using iodobenzene and phenyl boronic acid and the coupling product (biphenyl) was obtained within 35 min with a TOF value of 389 h^−1^. The enhanced catalytic performance may be attributed to the mono-dispersity and ultra-small size of Pd nanoparticles. The abundant hydroxyl groups on the surface of La(OH)_3_ nanorods and large surface area as evident by FTIR and BET calculations also favors the adsorption of reactants and offers plenty of anchoring sites for Pd ion and subsequently immobilizing Pd NPs resulting in the confinement of NPs and minimizing aggregation. The strong electronic communication between Pd nanoparticle and La(OH)_3_ nanorods ascertained by HRTEM and XPS analysis act as active catalytic centers for enhanced performance. Our study provides a deep insight into the design and development of highly efficient and low-cost catalyst for organic conversions.

## Experimental

### Chemicals

Lanthanum(III) nitrate hexahydrate (La(NO_3_)_2_·6H_2_O), polyvinyl pyrrolidone (PVP) (M.W. 8000), Ammonia solution (25%), sodium borohydride (NaBH_4_), iodobenzene, phenylboronic acid, K_2_CO_3,_ 4-nitrophenol (reagent grade), and styrene were obtained from Sinopharm Chemical Reagent and used without further purification. Palladium(II) chloride (PdCl_2_) were bought from Aldrich and used as received. Water was purified using ion exchange (MilliQ, Millipore), and served as deionized water.

### Characterization

Powder X-ray diffraction (XRD) measurements of the products were performed using a Philips X’Pert Pro Super X-ray diffractometer equipped with graphite monochromatized Cu Kα radiation (λ = 1.54178 A). Scanning electron microscopy (SEM) images were taken on a field-emission scanning electron microanalyzer (JEOL JSM-6700F, 15 kV). Transmission electron microscopy (TEM), and high-resolution TEM (HRTEM) images were recorded on a JEM-ARF200F transmission electron microscope. The high-angle annular dark-field scanning transmission electron microscopy (HAADF-STEM) image and EDX mapping images were taken on a JEOL JEM-ARF200F atomic resolution analytical microscope. The X-ray photoelectron spectroscopy (XPS) was carried out on a PerkinElmer RBD upgraded PHI-5000C ESCA system. The UV-vis absorption spectra were measured with a Shimadzu UV-2510 spectrophotometer in the region of 250 to 550 nm. Gas chromatograms (GC) were carried out on Agilent HP-5, Gas Chromatograph with a SGE BP1 non-polar 100% dimethylpolysiloxane capillary column of (30 m × 0.32 mm × 0.25 μm) dimensions. The measurements for final products of styrene and Suzuki coupling reactions were performed for 1 minute at 70 °C and a ramp of 10 °C min^−1^ until 110 °C. The inductively coupled plasma atomic emission spectroscopy (ICP-AES) method was taken on a PerkinElmer Optima 8000 ICP-AES/ICP-OES spectrometer to measure the Pd content in Pd/La(OH)_3_. Nitrogen adsorption-desorption measurements were carried out at 77 K with a micromeritics ASAP 2000 system using Barret-Emmet-Teller (BET) calculations for the surface area. Fourier transform infrared (FTIR) spectra were measured using MAGNA-IR750 (Nicole instrument Co. USA) infrared spectrophotometer with KBr dilution. The zeta potential measurements of La(OH)_3_ nanorods were performed on Malvern ZEN3600.

### Synthesis of Lanthanum hydroxide nanorods

In a typical synthesis, Lanthanum(III) nitrate hexahydrate (La(NO_3_)_2_·6H_2_O) 2.165 g and PVP 1.11 g were added into 100 ml DI water, and stirred for 3 hours. Then, required amount of ammonia solution was added to the above solution drop wise until the solution became white. The white gel was then transferred to 150 ml autoclave and heated at 160 °C for 24 hours. After cooling down the autoclave at room temperature, the product was obtained by centrifugation and washed with water and ethanol three times. Finally the product was dried at 60 °C in a vacuum oven and further used.

### Synthesis of Pd nanoparticles supported on La(OH)_3_ nanorods

For the loading of Pd onto La(OH)_3_ nanorods, 100 mg of La(OH)_3_ nanorods were dispersed into 100 mL of water via sonication and vigorous stirring. Afterwards, 1 wt% H_2_PdCl_4_ solution were added into the dispersion and stirred for a while. Then, the mixture solution was placed under UV light beam with continues stirring at room temperature for about 15 minutes. The product was separated via centrifugation, washed thoroughly with distilled water and ethanol and dried in vacuum oven at 60 °C overnight.

### Catalytic reduction of 4-nitrophenol

The reduction of 4-nitrophenol (4-NP) to 4-aminophenol (4-AP) with NaBH_4_ as the reductant at room temperature was done by adding 4-NP (60 μL, 10 mM) to 7 mL of NaBH_4_ aqueous solution (0.1 M) and 1 mL of catalyst dispersion (0.1 mg mL^−1^). UV-vis spectrometry was used to monitor the reaction progress of 4-NP reduction by measuring the absorbance of the solution from 550 to 250 nm as a function of time. The rate constant of the reaction was obtained by a plot of *ln*(A_t_/A_0_) and reaction time. Here A_t_ and A_0_ represent the concentrations of 4-NP at time = t and time = 0, respectively, which can be obtained from the decrease of peak intensity at 400 nm with time. The recycling performance was performed following the same procedure as above.

### Catalytic hydrogenation of styrene

The catalytic activity of Pd/La(OH)_3_ for the hydrogenation of styrene to ethylbenzene was performed in absolute ethanol using H_2_ as the reductant at room temperature. Typically, 5 mg of catalyst, 1.5 mmol of styrene and 2 mmol of 1,3,5-trimethylbenzene (internal standard) was added into 10 mL ethanol, and the mixture was sonicated for five minutes. Then, the mixture was stirred at room temperature for desired time under a H_2_ flow rate of 40 mL min^−1^. The products were analyzed by a gas chromatograph equipped with a flame ionization detector (FID). The recycling experiments and the catalytic activity of bare La(OH)_3_ nanorods follows the same procedures.

### Catalytic Suzuki cross-coupling reaction

For the Suzuki cross-coupling reaction, 0.3 mmol of Iodobenzene or one of its derivative, 0.6 mmol of phenylboronic acid, 1 mmol of potassium carbonate and 5.5 mg of catalyst were added into 50 ml ethanol:water (8:2 volume ratio) reaction solvent. The mixture was then stirred at 65 °C and the temperature of the reactant solution was maintained by water bath. The conversions were determined by GC-MS using diphenyl ether as the internal standard.

## Electronic supplementary material

Supporting Information
