# Supplementary material for: Interfacial Phenomenon and Nanostructural Enhancements in Palladium Loaded Lanthanum Hydroxide Nanorods for Heterogeneous Catalytic Applications
Source: Sci Rep. 2018 Mar 12;8:4354. doi: 10.1038/s41598-018-22800-0 (PMC5847562; doi:10.1038/s41598-018-22800-0)
Supplement: Supplementary file 1 — Supporting Information [file 41598_2018_22800_MOESM1_ESM.doc]

**Supporting Information**

**Interfacial Phenomenon and Nanostructural Enhancements in Palladium Loaded Lanthanum Hydroxide Nanorods for Heterogeneous Catalytic Applications**

Ammar Bin Yousaf,^1,‡,*^ Muhammad Imran,^2,‡,*^ Muhammad Farooq,^3^ and Peter Kasak,^1,*^

^1^ Center for Advanced Materials, Qatar University, Doha 2713, Qatar

^2^ Hefei National Laboratory for Physical Sciences at Microscale, University of Science and Technology of China, Hefei, Anhui 230026, PR China

^3^ Department of Chemistry, University of Management and Technology, Lahore, 54000, Pakistan

^‡^These two authors contributed equally to this work.

Correspondence and requests for materials should be addressed to A. B. Yousaf, M. Imran & P. Kasak

*Email: [ammar@mail.ustc.edu.cn](mailto:ammar@mail.ustc.edu.cn) , [ammar.chemist18@gmail.com](mailto:ammar.chemist18@gmail.com) (A.B.Y), [imran345@mail.ustc.edu.cn](mailto:imran345@mail.ustc.edu.cn) (M.I) [peter.kasak@qu.edu.qa](mailto:peter.kasak@qu.edu.qa) (P.K)


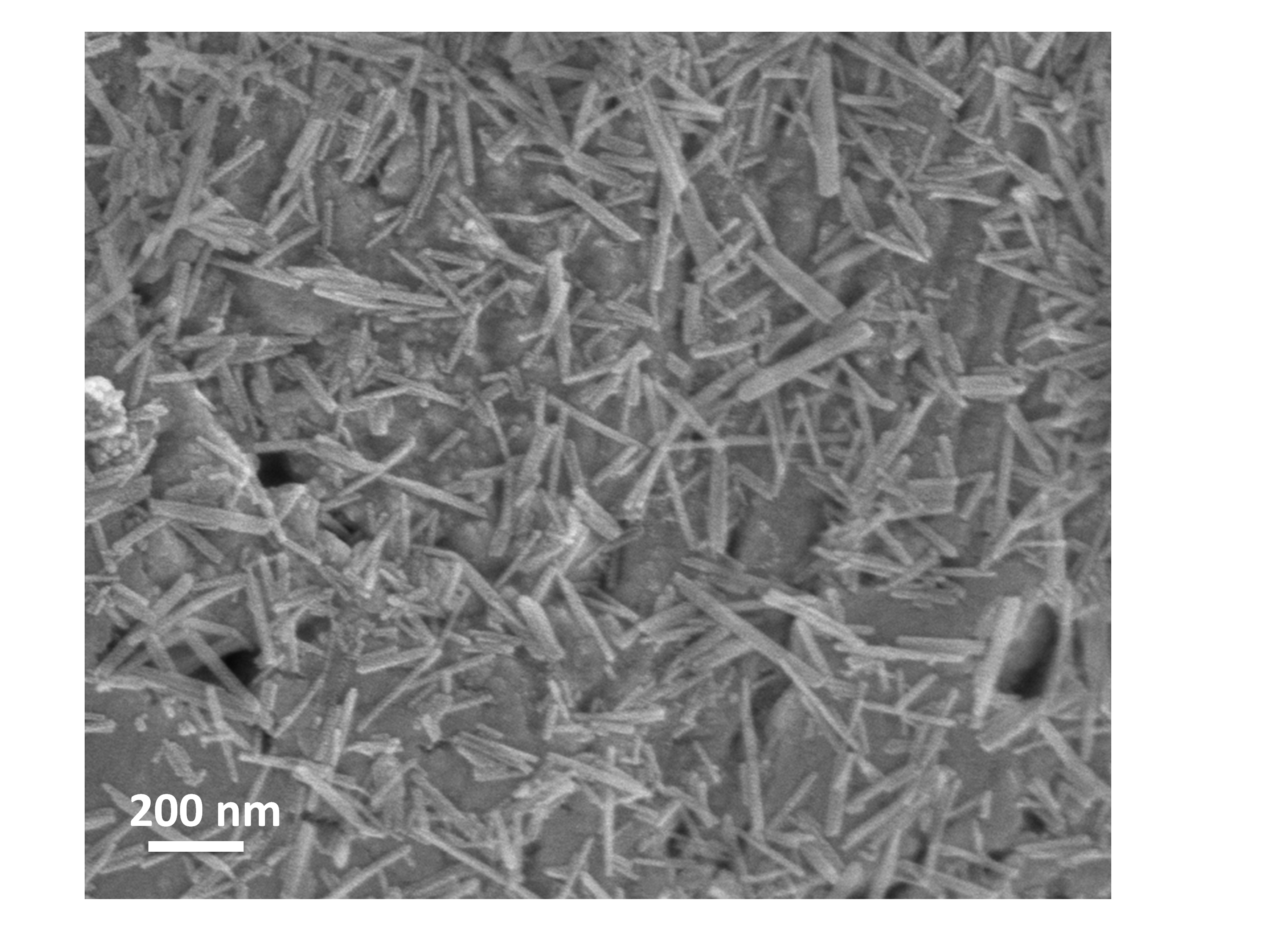


**Figure S1.** Scanning electron microscopy (SEM) of La(OH)_3_ nanorods.


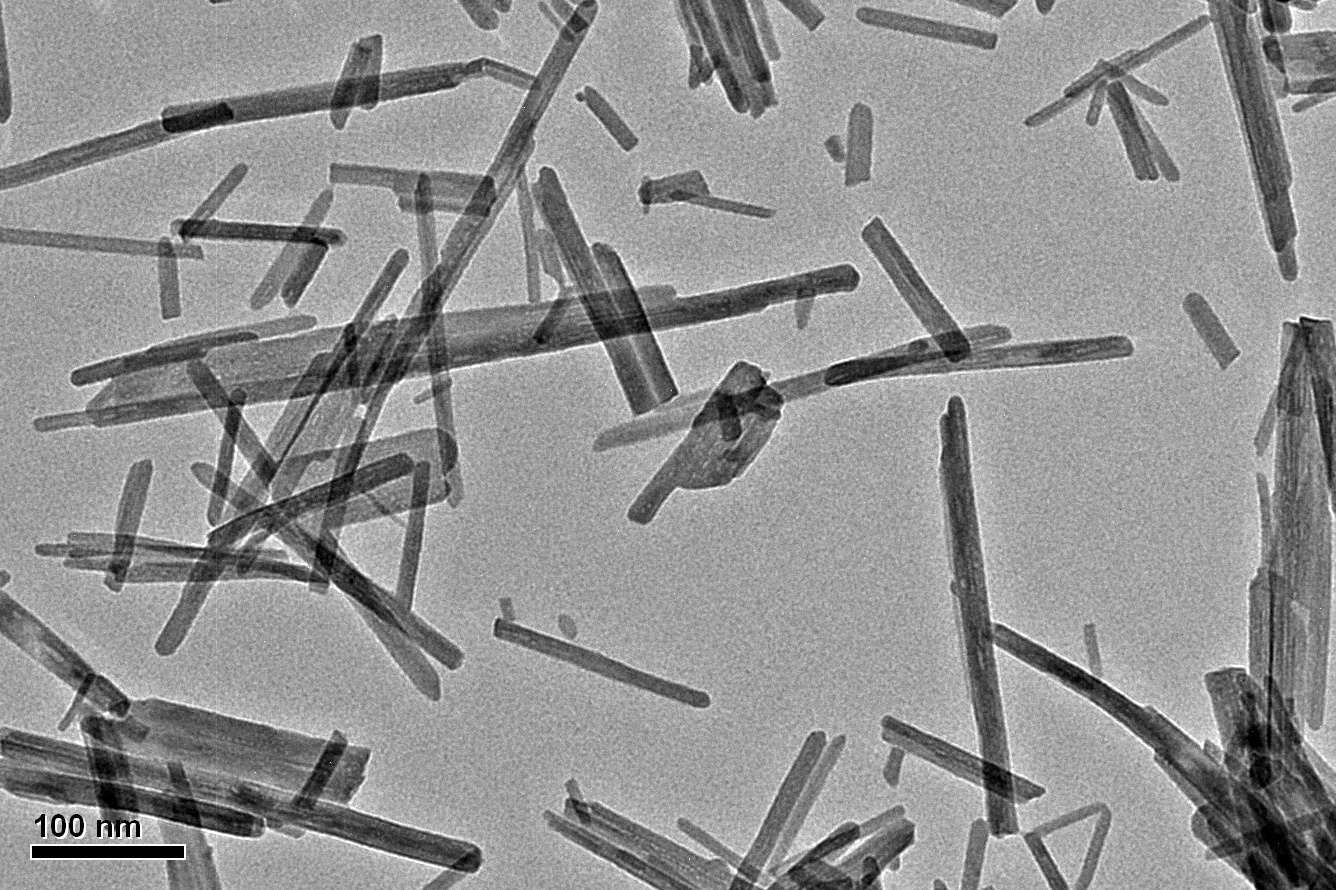


**Figure S2.** Transmission electron microscopy (SEM) of La(OH)_3_ nanorods.

**
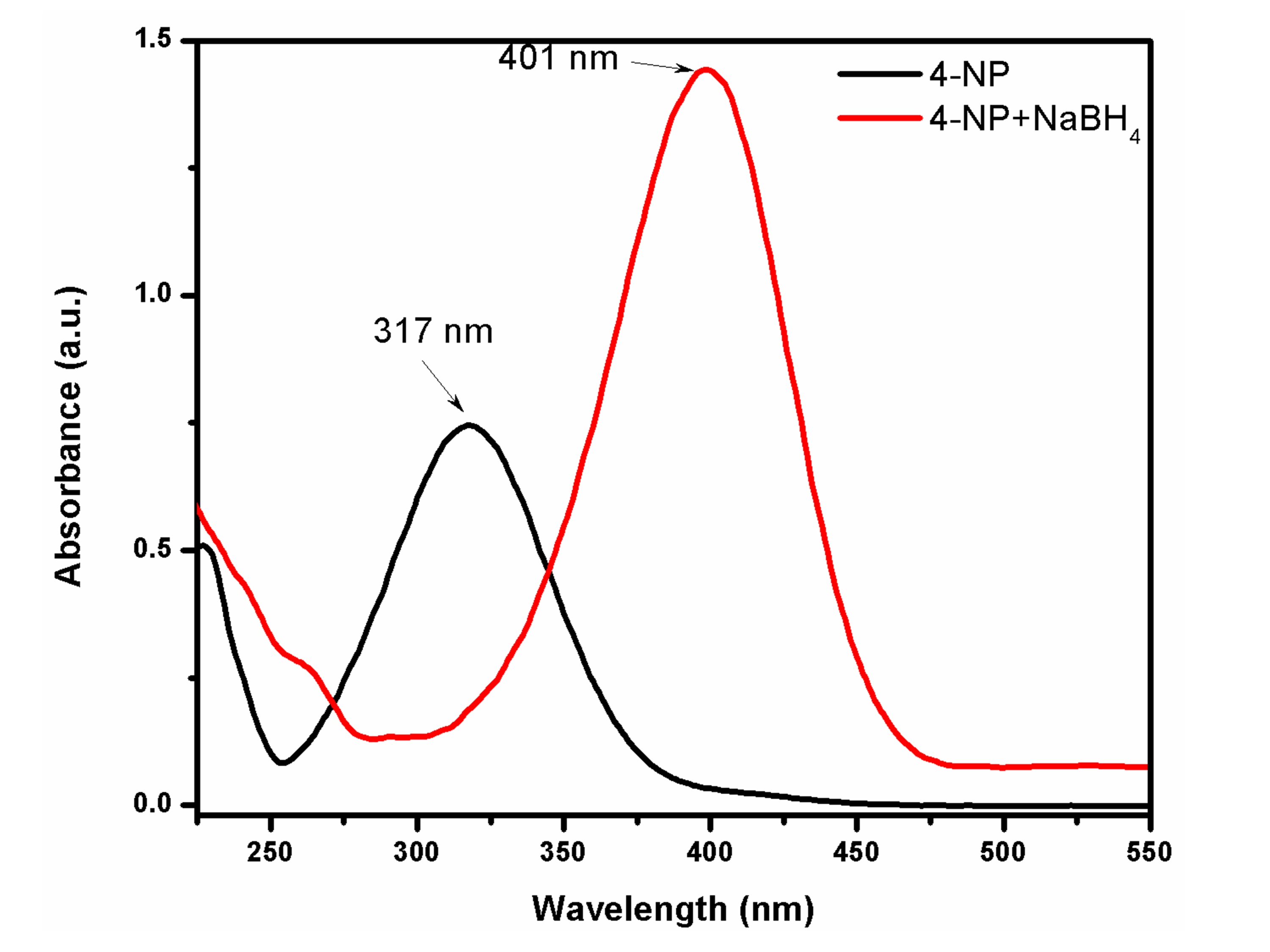
**

**Figure S3.** The absorption spectra of 4-nitrophenol (4-NP) with and without the addition of NaBH_4_.


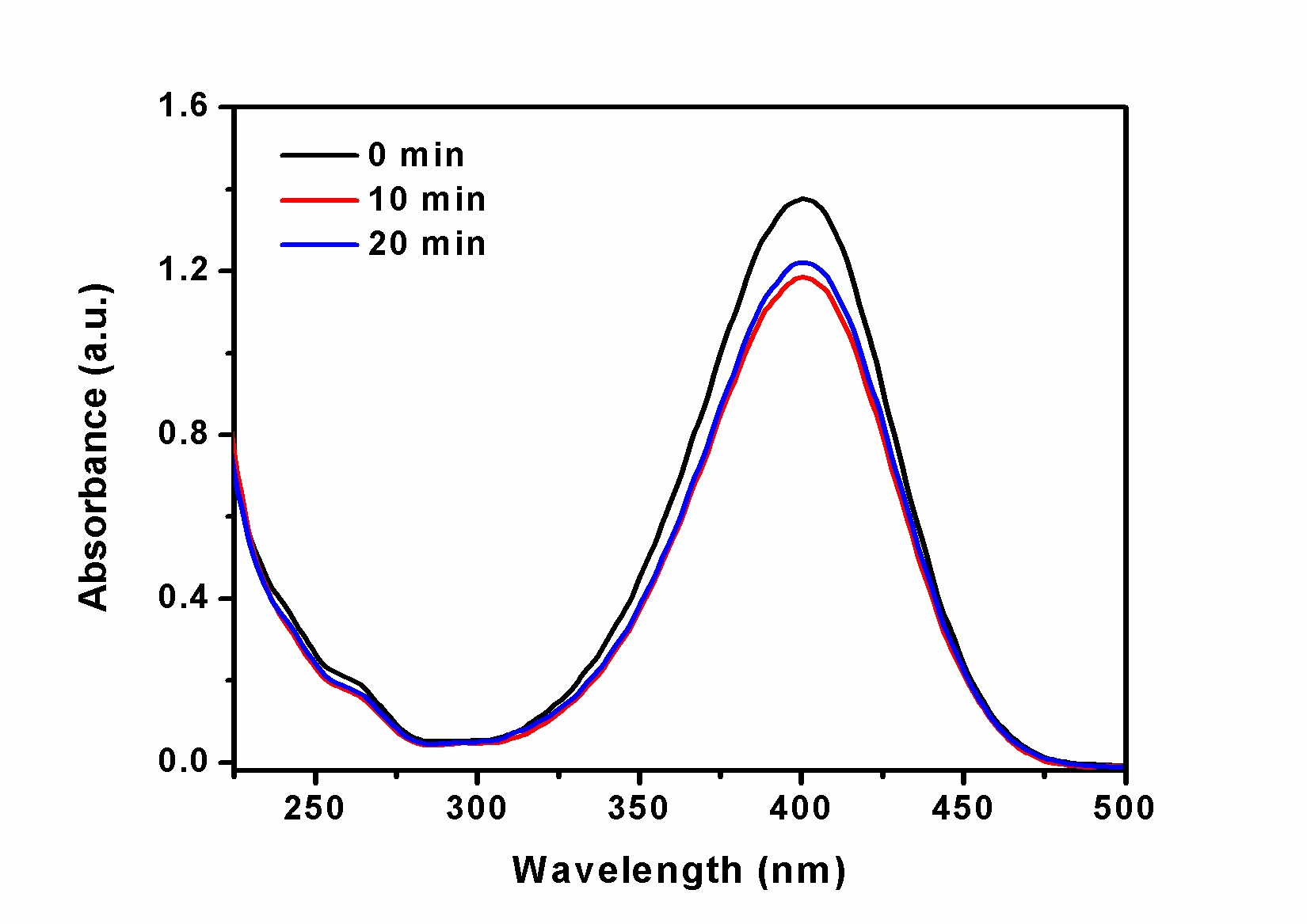


**Figure S4.** Time dependent UV-Visible spectra of 4-NP reduction over bare La(OH)_3_ nanorods.


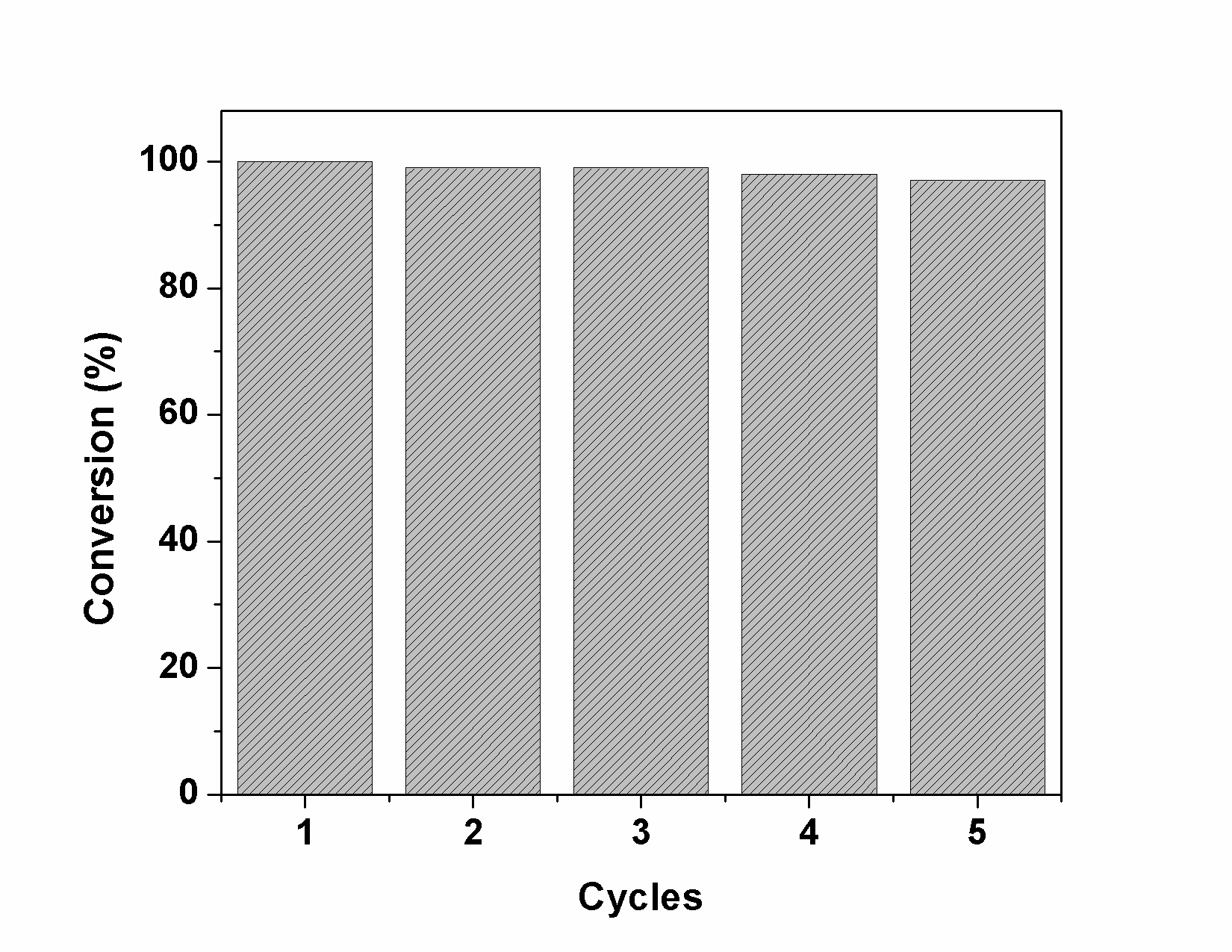


**Figure S5.** Recycling performance of Pd/La(OH)_3_ for 4-NP reduction.


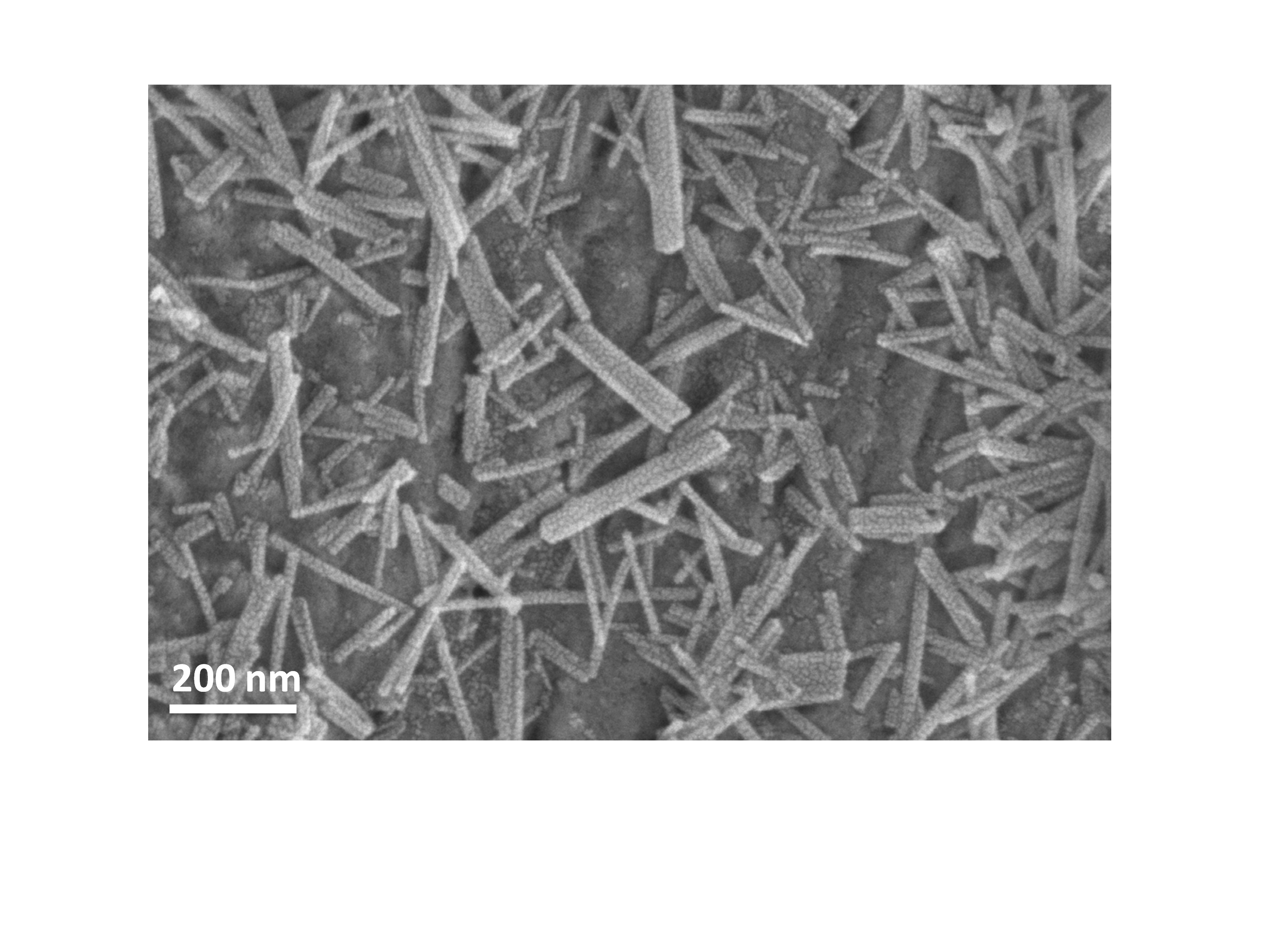


**Figure S6.** Scanning electron microscopy (SEM) of recycled Pd/La(OH)_3_ nanorods.


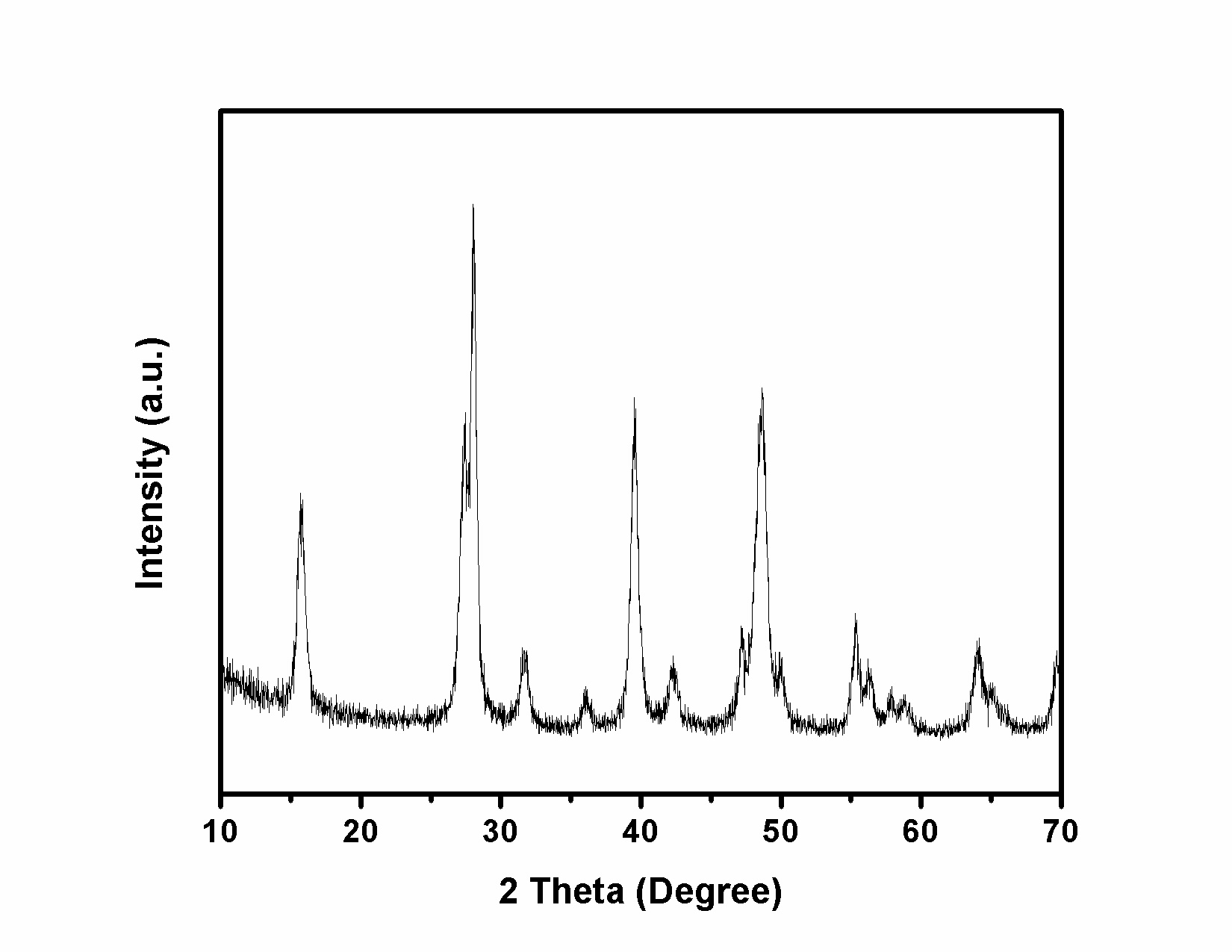


**Figure S7.** XRD patterns of recycled Pd/La(OH)_3_ nanocatalyst.

**Table S1: Comparison of catalytic reduction of 4-nitropehnol with reported data**

| Entry | Catalyst | *t (min)* | *k* (s^-1^) | Ref |
| --- | --- | --- | --- | --- |
| 1 | Pd/La(OH)_3_ | 0.75 (45s) | 60×10^-3^ | Our study |
| 2 | Au/PMMA | 10 | 7.9×10^-3^ | 1 |
| 3 | Pd@Y-DDQ | 3.3 | 17×10^-3^ | 2 |
| 4 | Pd/OCNTs | 4 | 16×10^-3^ | 3 |
| 5 | Pd-SPB | 20 | 4.4×10^-3^ | 4 |
| 6 | Pd-ZnO | 12 | 4.6×10^-3^ | 5 |
| 7 | Pd-CeO_2_ | 2 | 39×10^-3^ | 6 |
| 8 | Pd−Fe_3_O_4_ | 16 | 2.7×10^-3^ | 7 |
| 9 | Pd/Gd(OH)_3_ | 1 | 47×10^-3^ | 8 |

**Table S2: Comparison of hydrogenation of styrene to ethylbenzene over different Pd based catalysts.**

| Entry | Catalyst | TOF(h^-1^) | Ref |
| --- | --- | --- | --- |
| 1. 1 | Pd/La(OH)_3_ | 3260 | Our study |
| 1. 2 | Pd/C | 163 | 9 |
| 1. 3 | Pd/MOF-5 | 682 | 10 |
| 1. 4 | Pd/PEG | 660 | 11 |
| 1. 5 | Pd/ZIF-8 | 307 | 12 |
| 1. 6 | Pd nansheets | 454 | 13 |
| 1. 7 | Fe_3_O_4_-NC-PZS-Pd | 1792 | 14 |
|  | Pd/Gd(OH)_3_ | 6159 | 8 |

**Table S3.** The Pd content in Pd-La(OH)_3_ nanorods measured by inductively coupled plasma-atomic emission spectroscopy (ICP-AES).

| No. | Pd( µg/mL) | Percentage (%) |
| --- | --- | --- |
| 1 | 0.989 | 0.991 |
| 2 | 0.978 | 0.984 |
| 3 | 0.966 | 0.972 |
| Average |  | 0.982 |

**References**

1. Kuroda, K., Ishida, T., Haruta, M. Reduction of 4-nitrophenol to 4-aminophenol over Au nanoparticles deposited on PMMA. *J. Mol. Catal. A Chem.* **298**, 7–11 (2009).
2. Zhu, Y., Zhu, M., Xia, L., Wu, Y. L., Hua, H., Xie, J. M. Lanthanide Metal-Organic Frameworks with Six-Coordinated Ln(III) Ions and Free Functional Organic Sites for Adsorptions and Extensive Catalytic Activities. *Sci. Rep.* **6**, 29728 (2016).
3. Wang, C., Yang, F., Yang, W., Ren, L., Zhang, Y., Jia, X., Zhang, L., Li, Y. PdO nanoparticles enhancing the catalytic activity of Pd/carbon nanotubes for 4-nitrophenol reduction. *RSC Adv.* **5**, 27526–27532 (2015).
4. Mei, Y., Lu, Y., Polzer, F., Ballauff, M., Drechsler, M. Catalytic activity of palladium nanoparticles encapsulated in spherical polyelectrolyte brushes and core−shell microgels. *Chem. Mater.* **19**, 1062−1069 (2007).
5. Hu, Q. Y., Liu, X .W., Tang, L., Min, D., Shia, T. C., Zhang, W. Pd–ZnO nanowire arrays as recyclable catalysts for 4-nitrophenol reduction and Suzuki coupling reactions. *RSC Adv.* **7**, 7964–7972 (2017).
6. Du, C. H., Guo, Y., Guo, Y. L., Gong, X. Q., Lu, G. Z. Polymer- templated synthesis of hollow Pd−CeO2 nanocomposite spheres and their catalytic activity and thermal stability. *J. Mater. Chem. A* **3**, 23230−23239 (2015).
7. Tuo, Y., Liu, G. F., Dong, B., Zhou, J. T., Wang, A. J., Wang, J., Jin, R. F., Lv, H., Dou, Z., Huang, W. Y. Microbial synthesis of Pd/Fe_3_O_4_, Au/Fe_3_O_4_ and PdAu/Fe_3_O_4_ nanocomposites for catalytic reduction of nitroaromatic compounds. *Sci. Rep.* **5**, 13515 (2015).
8. Naseeb, U., Imran, M., Kuang, L., Yuan, C. Z., Akif, Z., Jiang, N., Umair, Y. Q., Shafaq, S., Xu, A. W. Highly dispersed ultra-small Pd nanoparticles on gadolinium hydroxide nanorods for efficient hydrogenation reactions. *Nanoscale* **9**, 13800−13807 (2017).
9. Hwang, C. B., Fu, Y.S., Lu, Y. L., Jang, S. W., Chou, P. T., Chris, W. C. R., Yu, J. S. C. Synthesis, Characterization, and Highly Efficient Catalytic Reactivity of Suspended Palladium Nanoparticles. *J. Catal.* **195**, 336−341 (2000).
10. Sabo, M., Henschel, A., Frode, H., Klemm, E., Kaskel, S. Solution Infiltration of Palladium into MOF-5: Synthesis, Physisorption and Catalytic Properties. *J. Mater. Chem.* **17**, 3827−3832 (2007).
11. Harraz, F. A., El-Hout, S. E., Killa, H. M., Ibrahim, I. A. Palladium Nanoparticles Stabilized by Polyethylene Glycol: Efficient, Recyclable Catalyst for Hydrogenation of Styrene and Nitrobenzene. *J. Catal.* **286**, 184−192 (2012).
12. Pan, Y., Ma, D., Liu, H., Wu, H., He, D., Li, Y. Uncoordinated carbonyl groups of MOFs as anchoring sites for the preparation of highly active Pd nano-catalysts. *J. Mater. Chem.* **22**, 10834− 10839 (2012).
13. Dai, Y., Liu, S. J., Zheng, N. F. C_2_H_2_ Treatment as a Facile Method to Boost the Catalysis of Pd Nanoparticulate Catalysts. *J. Am. Chem. Soc.* **136**, 5583−5586 (2014).
14. Yang, S. L., Cao, C. Y., Sun, Y. B., Huang, P. P., Wei, F. F., Song, W. G. Nanoscale Magnetic Stirring Bars for Heterogeneous Catalysis in Microscopic Systems. *Angew. Chem., Int. Ed.* **54**, 2661−2664 (2015).
